# Stromal collagen IV expression and risk of breast cancer death in ductal carcinoma in situ

**DOI:** 10.1038/s44276-025-00191-w

**Published:** 2025-10-21

**Authors:** Gunilla Rask, Malin Jansson, Johan Svensson, Rebecca Wiberg, Fredrik Wärnberg, Ola Billing, Charlotta Wadsten, Malin Sund

**Affiliations:** 1 Department of Diagnostics and Intervention, Surgery, Umeå University, Umeå, Sweden; 2 Department of Medical Biosciences, Pathology, Umeå University, Umeå, Sweden; 3 Department of Statistics, Umeå School of Business, Economics and statistics, Umeå University, Umeå, Sweden; 4 Department of Diagnostics and Intervention, Plastic Surgery, Umeå University, Umeå, Sweden; 5 Region Västra Götaland, Sahlgrenska University Hospital, Department of Surgery, Gothenburg, Sweden; 6 Department of Surgery, Institution of Clinical Sciences, Sahlgrenska Academy, University of Gothenburg, Gothenburg, Sweden; 7 Department of Surgery, University of Helsinki and Helsinki University Hospital, Helsinki, Finland

## Abstract

**Background:**

Current treatment for ductal carcinoma in situ (DCIS) of the breast is generic, due to lack of risk stratification tools. We investigate the correlation between expression of collagen IV in the breast and risk of dying of breast cancer. We also explore the effect of collagen IV in vitro.

**Methods:**

Tissue microarrays from a cohort of women treated for DCIS who later died from breast cancer (*n* = 43) or were still alive (*n* = 119), were analysed for collagen IV by immunohistochemistry. Oestrogen receptor positive (ER+), triple negative and human epidermal growth factor receptor 2 amplified (HER2+) cell lines were cultured with and without collagen IV.

**Results:**

High expression of stromal collagen IV correlated with increased odds of dying of breast cancer (OR 2.50; 95% CI 1.16–5.39). This association remained when adjusting for tumour size, margin status, comedo necrosis and progesterone receptor negativity (PR−) (OR 4.27; 95% CI 1.64–11.1).

Triple negative breast cancer cell lines migrated quicker on collagen IV-coated than on uncoated surfaces. By contrast, collagen IV coating did not affect ER+ and HER2+ cell lines.

**Conclusions:**

Abundance of stromal collagen IV increases risk of dying in breast cancer after DCIS, and collagen IV can promote cell motility in vitro.

## Background

As screening programmes are widely implemented and radiological methods become ever more refined, the proportion of women with breast cancer that are diagnosed with ductal carcinoma in situ (DCIS) is increasing and now comprises about 15% of new breast cancers [1]. Current treatment of DCIS is surgery with or without radiotherapy, with possible addition of endocrine treatment for women with oestrogen receptor positive (ER+) DCIS. As survival after DCIS treatment is excellent, there is rising concern that several women are overtreated, meaning that they would not have developed any symptomatic invasive carcinoma if left untreated [2]. Methods for robust risk stratification of patients is therefore in high demand.

Traditional histological factors, and those recommended in the EUSOMA (European Society of Breast Cancer specialists) guidelines are lesion size, nuclear grade, growth pattern, presence of comedo necrosis, calcifications and expression of ER [3]. Nuclear grade is at best however moderately reproducible between pathologists [4] and there are conflicting results as to whether grade, comedo necrosis, histological pattern and ER-status are related to patient outcome [5–9]. Assuming that old or novel biomarkers may be used to predict risk begs the question of which risk specifically. Is it risk of local DCIS recurrence, of invasive recurrence or of breast cancer death? All treatments come with side effects, and the benefit of risk reduction must be substantial enough to motivate treatment. Features which predict DCIS recurrence are not the same as those that predict recurrence as invasive disease [9]. Invasive recurrence increases risk of breast cancer death, and the addition of radiotherapy is routinely used to lower risk of local recurrence after breast conserving surgery. However, neither radiotherapy nor addition of endocrine therapy have been shown to affect the risk of breast cancer death [10–12].

To refine the traditional risk stratification of DCIS there is a need for new biomarkers that are related to the risk of distant metastasis and breast cancer death. Surrounding the DCIS cells is the myoepithelium, resting in turn on the basement membrane (BM) which is composed almost entirely of collagen IV. Outside of this structure is the stroma, normally consisting of many other types of collagens and smaller amounts of other molecules [13]. Cancer-associated stroma, however, often contains collagen IV also beyond the BM [14–16]. Our group has previously linked this stromal collagen IV expression to an increased risk of distant metastasis and worse survival [17]. Circulating collagen IV has also been linked to an increased risk of distant metastasis and worse survival in both breast [18] and colorectal [19] cancer patients. A previous study using mouse and in vitro models demonstrated that breast cancer cells of a predominantly triple-negative phenotype invade quicker and further in collagen IV-rich decellularized matrix scaffolds compared to scaffolds with low collagen IV-content [20].

This study aimed to determine 1) if stromal collagen IV-expression in DCIS correlated with risk of breast cancer death in a case-control cohort, and 2) if collagen IV could affect the motility of breast cancer cell lines in vitro.

## Materials and method

### Patient cohort and TMA-construction

**Fig. 1:**
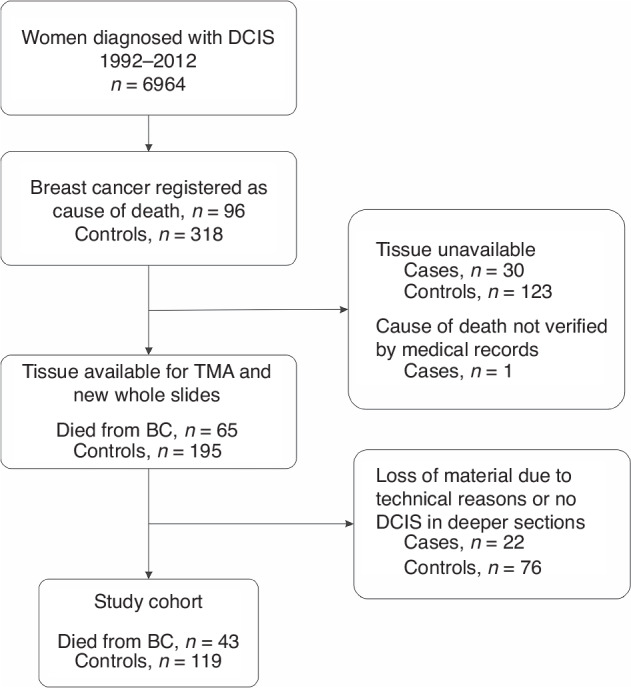
Selection of the study cohort. DCIS ductal carcinoma in situ, TMA tissue micro array, BC breast cancer.

We have previously described a nested case-control cohort of women with DCIS with clinicopathological data available [21]. Briefly, the cancer registries of three health care regions in Sweden were used to identify women treated for DCIS between 1992 and 2012 (*n* = 6 964). Cross-linking with the National Cause of Death Registry identified women who later died from breast cancer (cases, *n* = 95). Controls were selected using incidence density sampling, from the whole population of women with DCIS. Controls had to be alive and without distant metastasis of breast cancer at the time of death of the corresponding case (*n* = 318). Clinical data was gathered from medical records. In this cohort, tumour size larger than 25 mm and positive margin status were associated with increased risk of breast cancer death, while surgical treatment and radiotherapy were not. From the surgical specimens of the women in the cohort, tumour tissue was obtained for whole slide sectioning and construction of tissue microarrays (TMA) for 65 of the 95 cases (68%) and 195 of 318 controls (61%). Whole slides and TMA-slides were centrally evaluated by a specialized breast pathologist (G.R, blinded to outcome at time of scoring) for DCIS grade, presence of comedo necrosis, amount of tumour infiltrating lymphocytes (TILs), as well as expression of ER, progesterone receptor (PR), human epidermal growth factor receptor 2 (HER2) and proliferation marker Ki67 [22]. For the present study there was sufficient tumour tissue in the TMAs for analysis of collagen IV in 43 cases and 119 controls (Fig. 1).

### Immunohistochemistry for collagen IV

Tissue sections were stained with rabbit anti-collagen IV (AB748, Millipore, Billerica, United States) diluted 1:50 in blocking buffer for 2 h in room temperature. Sections were then washed in phosphate buffered saline (PBS) and incubated with the biotinylated secondary antibody, diluted 1:200 in blocking buffer, for 30 min in room temperature. Finally, slides were washed in PBS followed by addition of diaminobenzidine tetrahydrochloride (DAB) as chromogen.

Expression of collagen IV was scored separately in the compartment closest to the myoepithelial/stromal interface, defined as within 10 μm of the interface and dubbed periductal collagen IV, and in the rest of the stroma, henceforth called stromal collagen IV. Each compartment was scored on a scale 0–3 (0 = no, 1 = minimal, 2 = moderate and 3 = strong expression). Scoring was done by two independent researchers (G.R and M.J), blinded to outcome at the time of scoring. Discrepant cases were reviewed jointly, and a consensus score was set. There were one to six biopsies for each patient in the TMA, and in cases were the expression of collagen IV within a compartment differed between biopsies the maximum score was used for the final analyses.

Cases where only normal tissue was present in some or all of the biopsies were analysed if at least two such biopsies contained both ductal basal membrane and stroma. These scores were not used for the risk analyses.

### Cell cultures and migration experiments

Three different breast cancer cell lines were used. An ER+/HER2− cell line (MCF-7; ATCC HTB-22), an ER−/HER2− cell line (MDA-MB-231; ATCC HTB-26) and a ER−/HER2+ cell line (JIMT-1; RRID:CVCL_2077). Cells were cultured in Dulbecco’s Modified Eagle’s Medium (DMEM) 4,5% glucose with 10% foetal bovine serum (FBS) and 1% penicillin/streptomycin. For migration experiments 96-well plates were coated with either DMEM or collagen IV (ab7536 Abcam; 1 mg/mL) diluted in DMEM to a concentration of 29 ug/mL. Cells were seeded at a density of 5000 cells/well (MCF-7), or 2500 cells/well (MDA-MB-231 and JIMT-1). After seeding the cells were left to adhere for 4–6 h, then washed with PBS. New DMEM was added, and the plates were inserted to the Holomonitor M4, which uses quantitative phase imaging to automatically detect and measure live cells without labelling [23]. We used the adaptive Gaussian algorithm of detection to minimize background noise from the coating. Images were captured every 15 min for 24 h. In each experiment, six wells per breast cancer cell line were used, three with and three without collagen IV, respectively. Three fields of view were analysed for each well, each field of view containing ~50–200 cells. The speed of individual cells between each consecutive captured image frame (distance travelled/time between frames) was calculated by the built-in software of the HoloMonitor, as well as the average speed of all cells of each type between consecutive frames. The experiment was repeated thrice and the values of the resulting averages for all experiments were used for the final analyses. After all experiments were finished, all three cell lines tested negative for potential contamination of various Mycoplasma species.

### Statistical analysis

Odds ratios (OR) for dying of breast cancer in the nested case control cohort were calculated using conditional logistic regression and all analyses adjusted for time at risk. The following clinical variables were used: Age, tumour size (<25 mm or ≥25 mm, as in previous studies on this cohort [21]), margin status (negative vs positive, uncertain or missing), mode of detection (screening vs non-screening) and surgical treatment (breast conserving therapy only (BCS), BCS with radiotherapy (BCS + RT) or mastectomy). Moreover the following biomarkers were used: Nuclear grade according to Holland [24], comedo necrosis (absent vs. present) and ER and PR expression (positive if ≥10% of nuclei stained, in keeping with national guidelines for invasive carcinoma) [25]. Periductal TILs were categorized as 0–5%, 10% or ≥20% according to international guidelines [26]. The expression of both stromal and periductal collagen IV as described above was simplified to a two-grade scale for the final analyses with 0–1 termed “low” and 2–3 termed “high”. Pearson Chi-square was used to test for independence between variables. All expected counts were greater than five. The test for interaction between ER-status and outcome was conducted by adding an interaction term in the conditional logistic regression model.

Loss of material in sectioning resulted in missing values of the case response variable, making some strata entirely unused. To maximise data use, strata with cases treated at similar timepoints were fused. This gave a total of 32 strata, compared to the original 66. For the adjusted analyses, missing values for variables other than either collagen IV variable were imputed using multiple imputation in 20 datasets estimated with chained equations. For the imputation model, the variables in the conditional logistic regression models were used, including the responses periductal collagen IV and stromal collagen IV as well as time at risk and year of diagnosis. Proportion of imputed values were 16% for tumour size, 6% for ER-status and less than 5% for all other variables.

For comparison of means of average migration speed in the in vitro experiments, independent samples *t*-test was used. The distribution was somewhat skewed, but the sample sizes were large enough (all samples >150 observations) to support the use of the *T*-test due to the central limit theorem.

Conditional logistic regression analyses, imputation and tests for interaction were performed in StataIC15.1 and all other analyses in SPSS® version 23 (IBM, Armonk, NY, USA).

## Results

### Study population

Median age at diagnosis was 54 years, with no significant difference between cases and controls. A majority of the women (63.6%) were diagnosed within the breast cancer screening programme. Most were treated with breast conserving surgery (BCS) with or without subsequent radiotherapy (27.8% and 32.7% respectively) while 39.5% underwent mastectomy. None received endocrine therapy, in keeping with national guidelines. The proportion of tumours <25 mm was approximately equal to those ≥25 mm (43.8% and 40.1%, with missing data on 16%). Approximately 10% of the surgical specimens had resection margins that were either positive or uncertain.

### Risk factors for breast cancer death

Several characteristics associated with increased risk of breast cancer death in the unadjusted analysis: Tumour size ≥25 mm (OR 3.48; 95% CI 1.42–8.56), presence of comedo necrosis (OR 2.68; 95% CI 1.10–6.53), negative PR expression (OR 2.45; 95% CI 1.08–5.57), and high expression of stromal collagen IV (OR 2.50; 95% CI 1.16–5.39).

Neither grade nor ER-expression had a statistically significant correlation with risk of breast cancer death. Table 1 summarizes the characteristics of the study cohort and ORs for breast cancer death.

**Table 1:** Clinicopathological factors and odds ratios for breast cancer death after treatment for ductal carcinoma in situ.

Clinicopathological variables	Study cohort (*n* = 162)	Cases (*n* = 43)	Controls (*n* = 119)	OR (95% CI) for breast cancer death^a^
Age yrs, median (IQR)	54 (48–62)	52 (46–62)	54 (49–62)	0.99 (0.96–1.02)
Tumour size^b^ *n* (%)				
<25 mm	71 (43.8)	11 (25.6)	60 (50.4)	1.0 (ref.)
≥25 mm	65 (40.1)	24 (55.8)	41 (34.5)	3.48 (1.42–8.56)
Missing	26 (16.0)	8 (18.6)	18 (15.1)	–
Margin status *n* (%)				
Negative	145 (89.5)	34 (79.1)	111 (93.3)	1.0 (ref.)
Positive/uncertain/missing	17 (10.5)	9 (20.9)	8 (6.7)	2.72 (0.94–7.87)
Mode of detection *n* (%)				
Screening	103 (63.6)	22 (51.2)	81 (68.1)	1.0 (ref.)
Non-screening	50 (30.9)	14 (32.6)	36 (30.3)	1.50 (0.68–3.32)
Missing	9 (5.6%)	7 (16.3)	2 (1.7)	–
Breast surgery *n* (%)				
BCS	45 (27.8)	12 (27.9)	33 (27.7)	1.0 (ref.)
BCS + RT	53 (32.7)	12 (27.9)	41 (34.5)	0.86 (0.33–2.25)
Mastectomy	64 (39.5)	19 (44.2)	45 (37.8)	1.31 (0.55–3.18)
Grade *n* (%)				
1	26 (16.0)	5 (11.6)	21 (17.6)	1.0 (ref.)
2	78 (48.1)	23 (53.5)	55 (46.2)	1.84 (0.63–5.33)
3	55 (34.0)	14 (32.6)	41 (34.5)	1.58 (0.49–5.10)
Missing	3 (1.9)	1 (2.3)	2 (1.7)	–
Comedonecrosis^b^ *n* (%)				
Absent	51 (31.5)	8 (18.6)	43 (36.1)	1.0 (ref.)
Present	108 (66.7)	34 (79.1)	74 (62.2)	2.68 (1.10–6.53)
Missing	3 (1.9)	1 (2.3)	2 (1.7)	–
ER *n* (%)				
≥10%	112 (69.1)	27 (62.8)	85 (71.4)	1.0 (ref.)
<10%	41 (25.3)	13 (30.2)	28 (23.5)	1.63 (0.73–3.67)
Missing	9 (5.5)	3 (7.0)	6 (5.0)	–
PR^b^ *n* (%)				
≥10%	93 (57.4)	20 (46.5)	73 (61.3)	1.0 (ref.)
<10%	63 (38.9)	21 (48.8)	42 (35.3)	2.45 (1.08–5.57)
Missing	6 (3.7)	2 (4.7)	4 (3.3)	–
Periductal TILs *n* (%)				
0–5%	98 (60.5)	23 (53.5)	75 (63.0)	1.0 (ref.)
10%	31 (19.1)	6 (14)	25 (21)	0.83 (0.30–2.32)
≥20%	30 (18.5)	13 (30.2)	17 (14.3)	2.34 (0.95–5.76)
Missing	3 (1.9)	1 (2.3)	2 (1.7)	–
Stromal collagen IV^b^ *n* (%)				
Low (0–1)	110 (67.9)	23 (53.5)	87 (73.1)	1.0 (ref.)
High (2–3)	50 (30.8)	19 (44.2)	31 (26.1)	2.50 (1.16–5.39)
Missing	2 (1.2)	1 (2.3)	1 (0.8)	–
Periductal collagen IV *n* (%)				
Low (0–1)	81 (50.0)	17 (39.5)	64 (53.8)	1.0 (ref.)
High (2–3)	81 (50.0)	26 (60.5)	55 (46.2)	2.00 (0.96–4.15)

*OR* odds ratio, *ER* oestrogen receptor, *PR* progesterone receptor, *TILs* tumour infiltrating lymphocytes.

^a^All analyses adjusted for time at risk.

^b^Denotes variables with statistically significant difference between cases and controls.

In the present cohort, a positive or uncertain margin status showed a non-significant trend towards increased odds for breast cancer-related death (OR 2.72; 95% CI 0.94–7.87). The dropout analysis showed fewer small (<25 mm) tumours remaining in the present cohort compared to tumours not analysed for collagen IV (43.8% vs 70.2%). The remaining cases were in all other respects similar to the cases that were lost. By contrast, the remaining controls were less often screening detected (68.1% vs 76.1%), had fewer negative surgical margins (93.3% vs 98.9%), more often had a suspicion of microinvasion (7.6% vs 0.99%) and were more often treated by mastectomy (37.8% vs 25.1%) than the controls that could not be analysed for collagen IV. Comparisons between the original cohort and the present one are summarised in Supplementary Table S1.

### Collagen IV expression in normal tissue

**Fig. 2:**
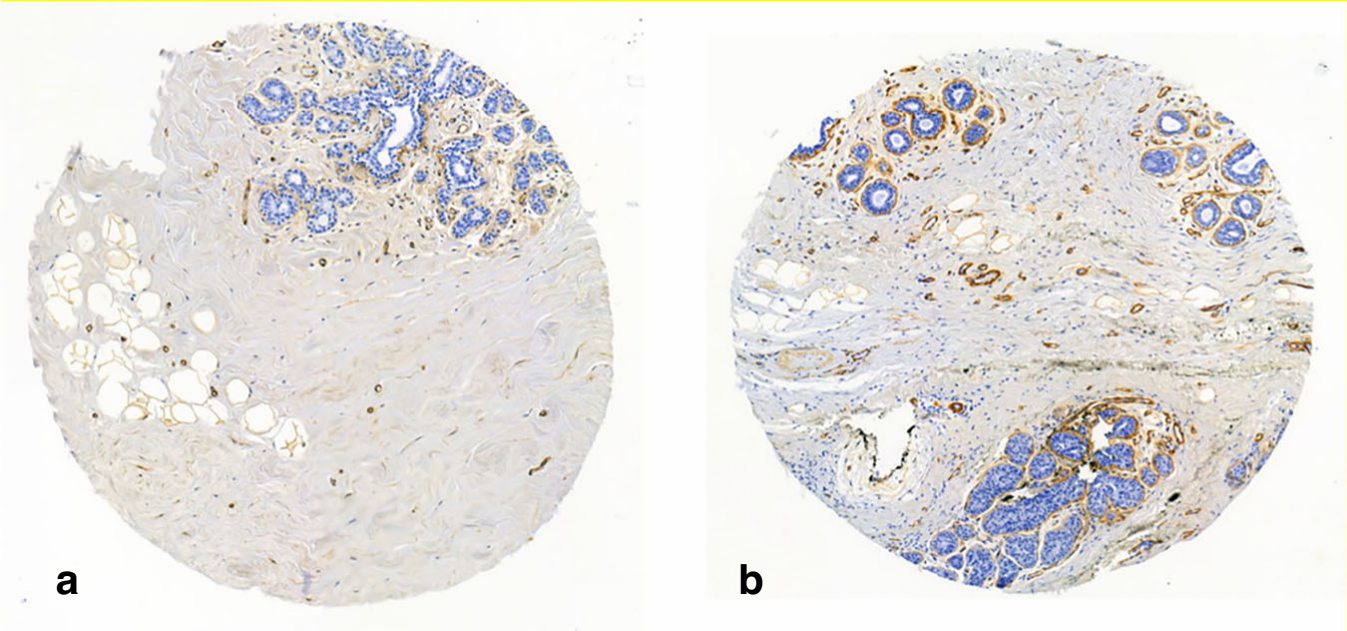
Examples of most common collagen IV staining patterns in normal tissue. **a** Periductal score 1 and stromal score 1; **b** Periductal score 2 and stromal score 1.

There were 17 cases with assessable periductal and stromal structures in at least two biopsies containing only normal tissue. These typically showed crisp, sometimes delicate periductal staining, and low stromal staining; only two cases had moderate (=2) stromal staining in one biopsy each. There was no negative (=0) periductal staining and no intense (=3) stromal staining. Neither were there any cases where the stromal staining exceeded the periductal staining. There was little periductal heterogeneity with three cases showing varying periductal staining between biopsies (0 and 1; 1 and 2; 2 and 3) while stromal staining varied between 0 to 1 for four cases and 1 to 2 for the two cases mentioned above. The results are summarized in Supplementary Table S2 and examples of staining are shown in Fig. 2.

### Collagen IV expression in DCIS and relation to other clinicopathological variables

**Fig. 3:**
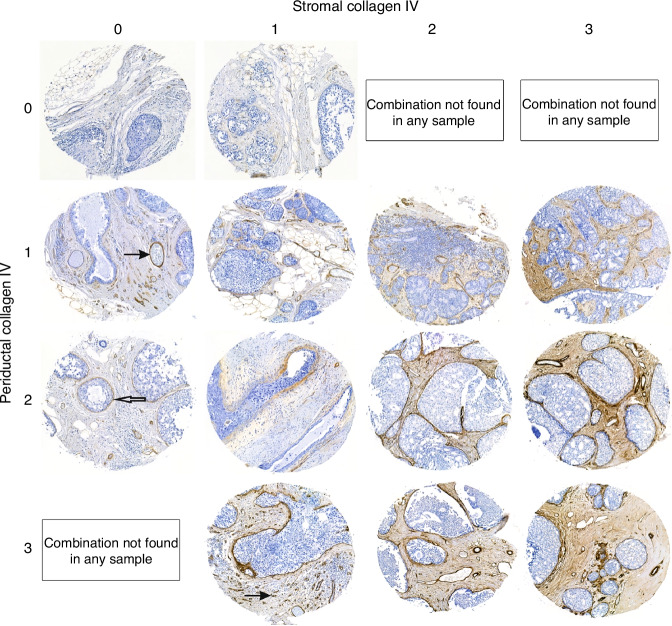
Examples of collagen IV staining in DCIS. Open arrow – periductal collagen IV, filled arrows – normal collagen IV in blood vessels; DCIS ductal carcinoma in situ.

Interobserver agreement between the two assessors for collagen IV expression in the TMA-biopsies, when using the 0 to 3 scale, was moderate for both periductal (kappa 0.52) and stromal (kappa 0.43) expression. Distinguishing between 0 and 1 for the stromal component, and 2 and 3 for the periductal component was perceived as hardest for both assessors. The number of tumours with stromal score 3 was low (*n* = 9 (5%). Grouping the variables as low (0 or 1) and high (2 or 3) gave substantial interobserver agreement, with kappa 0.65 for stromal and 0.76 for periductal expression, as well as increasing statistical power. Representative images of TMA-biopsies with different expression of collagen IV are shown in Fig. 3, and original four-grade scores for cases and controls are shown in Supplementary Table 3.

There was an association between expression of periductal and stromal collagen (*p *< 0.001) i.e. they were not independent variables. There was no dependency between collagen IV expression and ER or PR expression. There was a borderline significant association between comedo necrosis and stromal collagen IV (*p *= 0.06), where DCIS with necrosis was more likely to have a low stromal collagen expression (73% vs 58%). Necrosis did not correlate with periductal collagen IV (*p *= 0.73). There was no statistically significant association between stromal collagen IV and tumour size (*p *= 0.08). However, smaller tumours (<25 mm) trended towards higher stromal collagen IV expression (39% vs. 20%).

There were 101 cases (62%) with more than one biopsy with assessable periductal staining. Forty-three of these (43%) showed any kind of heterogeneity between biopsies, but only 18 (18%) varied across the “high” and “low” categories, and there was only one case showing variation from score 1 to 3, the other 17 varied from 1 to 2. The stromal staining showed overall heterogeneity at the same level, with 41 of 98 cases (42%). Variation across the “high” and “low” categories (25/98; 26%) was somewhat more common, and four cases had variation across the spectrum of 1 to 3. Accordingly, only ten of 35 cases having more than one biopsy and categorized as “high” had high stromal staining in all biopsies, while 25 of 35 showed high staining only in some of the biopsies. Presence of heterogeneity did not correlate with risk of death within this group (*p *= 0.68).

### Collagen IV expression predicts breast cancer-related death in adjusted models

In the previous unadjusted analysis, high stromal collagen IV expression correlated with increased odds of dying of breast cancer. This association remained significant when adjusting for tumour size and margin status (OR 3.62; 95% CI 1.45–9.00), and even after adjusting for presence of comedo necrosis and PR negativity (OR 4.27; 95% CI 1.64–11.1). Subgroup analysis of ER-positive and ER-negative tumours could not be performed due to small sample size, but the interaction test did not show any interaction between ER and stromal collagen IV for the risk of breast cancer death (*p *= 0.19). Similarly, the number of patients with positive or uncertain margins was too small for either subgroup analysis or interaction testing, but analysis of only patients with clear margins showed essentially similar correlation between stromal collagen IV and odds of breast cancer death as in the whole cohort (OR 3.21; 95% CI 1.30–7.90).

Periductal expression of collagen IV was not statistically significantly associated with increased odds of breast cancer death in the unadjusted analysis (OR 2.00; 95% CI 0.96–4.15). However, it showed a similar association as stromal collagen IV when adjusting for tumour size and margin status (OR 2.50; 95% CI 1.10–5.64) as well as with further adjusting for comedonecrosis and PR (OR 2.55; 95% CI 1.11–5.86). The results are summarized in Table 2.

**Table 2:** Collagen IV expression and risk of breast cancer death.

	OR for breast cancer death (95% CI)^a^		
	Unadjusted	Adjusted for tumour size and margin status	Adjusted for tumour size, margin, PR and necrosis
Stromal collagen IV			
Low (0-1)	1.0 (ref.)	1.0 (ref.)	1.0 (ref.)
High (2-3)	2.50 (1.16–5.39)	3.62 (1.45–9.00)	OR 4.27 (1.64–11.1)
Periductal collagen IV			
Low (0-1)	1.0 (ref.)	1.0 (ref.)	1.0 (ref.)
High (2-3)	2.00 (0.96–4.15)	2.50 (1.10–5.64)	2.55 (1.11–5.86)

*OR* odds ratio, *CI* confidence interval, *PR* progesterone receptor.

^a^All analyses adjusted for time at risk.

Using the original four-grade scoring system, increased odds for breast cancer death were observed across all collagen IV categories compared to category 0 in the unadjusted analyses. However, statistical significance was only reached for stromal collagen IV score 2 (OR 4.00, 95% CI 1.17–13.69), with generally wide confidence intervals due to the small sample size. Results are presented in Supplementary Table 4

### Collagen IV and characteristics of recurrent disease

There were in total 38 known invasive in-breast recurrences (ipsi- and contralateral) in the case group, whereof 15 (63% of those with known ER− status) were ER+ and four (31% of those with known HER2-status) were HER2+. In the control group there were 10 invasive recurrences, whereof five of seven with ER-status available (71%) were ER+ and none were HER2+. For DCIS with low stromal collagen IV, ten of 16 (63%) of recurrences with known ER-status were ER+, as were ten of 15 (67%) recurrences after DCIS with high stromal collagen IV. HER2-positive recurrences numbered four (44%) in the low stromal collagen IV group, and none in the high stromal collagen IV group. A summary of recurrences are shown in Supplementary Table S5a, b.

### Collagen IV and cell migration

**Fig. 4:**
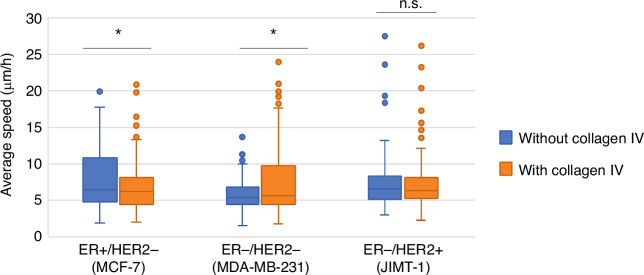
Migration speed per cell type with and without collagen IV coating. Mean speed of all cells of each subtype between each consecutive recorded image. ER oestrogen receptor, HER2 human epidermal growth factor receptor 2; Asterisk (*) denotes significantly different mean average migration speed (*p *< 0.001); n.s. not statistically significant.

As collagen IV has been shown to increase motility in vitro only in triple negative but not other types of breast cancer cells, experiments were performed using triple negative, ER+/HER2− and ER−/HER2+ cell lines. By light microscopy, the cells displayed different phenotypes. The triple negative cells (MDA-MB-231) were elongated and mesenchymal-like, growing evenly dispersed across the plates. The ER+ and ER−/HER2+ cells (MCF-7 and JIMT-1) were rounder, plumper and tended to grow in aggregates rather than spreading out. The appearances were the same in both collagen IV-coated and uncoated plates. Effects of collagen IV-coating on cell migration differed between cell types (Fig. 3). Collagen IV coating resulted in an increased average migration speed in triple negative cells (5.7 vs. 7.7 μm/h, *p *< 0.001), whereas the opposite was observed for ER+/HER2− cells (7.8 vs. 6.6 μm/h, *p *< 0.001). No effect on migration speed was seen in the ER−/HER2+ cell line (7.3 vs. 7.1 μm/h, *p *= 0.68) (Fig. 4).

## Discussion

Stromal components and other factors related to the tumour microenvironment are gaining increasing attention as potential prognostic markers and treatment targets in many cancer types [27]. We demonstrate for the first time that high levels of collagen IV, both in the stromal and periductal compartment in DCIS are associated with increased odds of breast cancer death. This association became even more pronounced when adjusting for established clinical risk factors, such as tumour size and positive margins, as well as tentative histological risk factors such as comedo necrosis and PR-negativity. Moreover, we confirm previous findings that collagen IV promotes migration of triple negative breast cancer cells [20], but the same was not shown for either ER+/HER2− or ER−/HER2+ breast cancer cells.

In the breast, collagen IV is normally secreted by the myoepithelial cells (MECs), but in invasive breast cancer the collagen IV in the extracellular matrix (ECM) can originate from the cancer cells themselves, the surrounding stromal cells or both [28]. In DCIS, production only by the cancer cells seems less likely, since the MEC layer limits physical access to both periductal and stromal compartments. Considering the MECs, it is known that tumour associated MECs differ in many ways from native MECs in gene expression patterns and phenotype [29–31]. To the best of our knowledge though, an increased production of collagen IV by cancer associated MECs has not yet been demonstrated.

That at least part of the collagen IV is produced by stromal cells is highly likely. Collagen IV self-polymerizes and cannot diffuse easily [32] but our analyses show stromal collagen IV expression as far as one millimetre from the DCIS (the limit of the diameter of our TMA-cores). If it is produced in the stroma, it also means that direct contact between DCIS cells and collagen IV-producing cells is not feasible as the only mode of communication. Either the signal needs to be endocrine rather than paracrine, or the DCIS cells also need to inform the stromal cells to feed the signal for increased collagen IV-production forward. It has been shown that the DCIS-induced PDGFRα^(low)^/PDGFRβ^(high)^ phenotype of stromal fibroblasts is dependent on direct cell-to cell or paracrine signalling [33]; investigating the levels of collagen IV in healthy breast tissue several centimetres away from the DCIS could show if the same is true for collagen IV-expression. The heterogeneity between biopsies in our material however, in particular for the stromal expression, seems to indicate that there is at least some local effect, rather than widespread increase throughout the breast. This is supported by previous studies on whole tissue slides showing increased expression of stromal collagen IV only in close proximity to neoplastic cells in pancreatic cancer [34], cholangiocarcinoma [35] and colon cancer [36]. All together this supports the notion that the stromal effect is local and not systemic. A radical excision of the tumour area would then also remove the collagen IV-rich stroma, leading to two possible explanations for the observation that high collagen IV expression correlates with breast cancer death. Either it may be a marker of DCIS with potential for disseminating tumour cells into the blood stream in the absence of microscopically detected invasion, leading to invasive recurrence – or it is a marker of a host stroma that is more susceptible to cancer induced stromal changes that facilitate invasion and metastasis. In the latter case, if the same patient suffers a recurrence, the new cancer cells would just as easily induce the same stromal changes as the previous DCIS. Studying the stroma of paired primary breast tumours and recurrences would elucidate this question, unfortunately we did not have access to tissue from recurrent tumours. Supporting the notion of collagen IV as at least partly a marker of host susceptibility is a study showing obese mice increase the collagen IV content of their ECM, with faster tumour development and more metastases than lean mice. Also, triple negative breast cancer cells migrate quicker and further in decellularized scaffolds from obese mice than lean mice [37].

Regardless of origin and distribution, the question of why increased collagen IV would lead to increased risk of breast cancer death remains unclear. Almost all in vitro or animal models exploring the effects of collagen IV have used triple negative cell lines [20, 38, 39], and all show increased migration in response to collagen IV exposure. One study also found similar results in non-neoplastic mammary epithelial cells [40] but effects on ER+ or HER2+ breast cancer cells have not been reported previously. Surprisingly, we found that a collagen IV coating decreased the migration speed of ER+/HER2− cells. This finding needs to be validated in additional cell lines and in other models. Cells may migrate by different means, either as single cells without any cell-to cell interaction, or as loosely or non-cohesive cell groups that move along the same path (streaming), or as cohesive units along a broad front (collective migration) [41]. One explanation to our findings may be that the mode of migration differs between the two cell types and that collagen IV, when used as coating, mainly increases one or two of these migration modes. Triple negative breast cancer cells (MDA-MB-231) migrate by single cell or streaming [41], while ER+ cells (MCF7) are less investigated also in this respect. We found no effect of collagen IV coating on the migration of ER−/HER2+ cells when looking at the sum of all experiments. This cell line was, however, somewhat more difficult to get good readings of in the HoloMonitor, which might have been due to the conformation of the cells or how they affected the coating during incubation, which is a limitation of these experiments.

Apart from local effects on cancer cell migration, increased collagen IV could also be one of the first steps in priming distant locations for metastasis. Previous work from our group has shown increased circulating collagen IV levels in patients with breast cancer and distant metastasis [18]. Such metastasis priming would also explain increased risk of cancer mortality, regardless of whether cell migration increases or not. One study showed that small extracellular vehicles secreted by tumour cells growing in a stiff, tumour associated ECM, contained abundant integrins, adhesion molecules and immune evasion signals, a combination that can induce a cancer-associated fibroblast phenotype in fibroblasts at distant sites. These vehicles more easily attached to, and stayed in, stroma that was rich in collagen IV, compared to vehicles from non-tumour associated stroma, which did not contain the same tumour promoting cargo [42]. As our study is retrospective we did not have the possibility of looking at blood collagen IV levels, presence of obesity or paired biopsies from metastatic and/or normal tissue sites to further investigate any correlations between these and the amount of collagen IV around the DCIS.

Other limitations of the present study include the use of TMA rather than whole tissue sections since the field of vision is limited to stroma within 1 mm from the DCIS. This prevents observation of collagen IV in healthy tissue further from the DCIS as well as observations regarding spatial heterogeneity in the tumour stroma. Looking at heterogeneity within those tumours with more than one biopsy, however, did not indicate that homogenous high expression was worse than high expression in only one or some biopsies.

Immunohistochemistry is a quick and relatively easy and cheap method of semi-quantitative analysis of the amount of any protein in histological sections. This is the reason why all clinically relevant biomarkers in surgical pathology currently are either IHC analyses or genetic analyses. The major drawbacks are that quantitative analysis is not possible and that there may be background, or unspecific, staining. That the samples with normal tissue in our cohort most commonly showed minimal stromal staining, when they should be devoid of collagen IV by our current understanding [13] is likely due to background staining, although it cannot be excluded that it is due to an effect of nearby DCIS. Lastly, the case-control cohort is small in numbers, preventing further subgroup analysis. This is however almost inevitable when dealing with rare outcomes such as breast cancer death after DCIS, and the cohort is in this respect larger or equal to others.

In conclusion our results indicate that stromal collagen IV correlates with risk of breast cancer death already at the in situ -stage of disease. This means that there may be aggressive types of DCIS with the potential to create conditions necessary for metastatic spread even before invasion. We further confirm that collagen IV increases migration of triple negative breast cancer cells, but not necessarily of any other type of breast cancer subtypes. Further studies should focus on collagen IV expression in healthy tissue of breast cancer patients as well as on how collagen IV affects different modes of migration.

## Supplementary information

REMARK-checklist-collagenIV

Table S1_original_vs_collagenIV

Table S3 coll IV

Table S4 coll IV

Tables S5a_S5b

Table S2

## Data Availability

Data is not uploaded to a publicly available platform. Researchers have access to data through application to the study PI (malin.sund@umu.se) or the corresponding author under standard rules of protecting data integrity and existing ethics permissions.
