# Supplementary material for: Stromal collagen IV expression and risk of breast cancer death in ductal carcinoma in situ
Source: BJC Rep. 2025 Oct 21;3:73. doi: 10.1038/s44276-025-00191-w (PMC12540875; doi:10.1038/s44276-025-00191-w)
Supplement: Supplementary file 2 — Table S1_original_vs_collagenIV [file 44276_2025_191_MOESM2_ESM.docx]

Table S1 Comparison of the 96 cases and 318 controls in the original study cohort with the 43 cases and 119 controls included in the present study

|  |  | Original cohort | | | |  | Cohort analysed for collagen IV | | | | p-value  (tumours analysed for collagen IV vs those that could not be analysed) |
| --- | --- | --- | --- | --- | --- | --- | --- | --- | --- | --- | --- |
|  |  | *Cases*  *n=96* | | *Controls*  *n=318* | |  | *Cases*  *n=43* | | *Controls*  *n=119* | |  |
| **Median age (yr, range)** |  | 54 | (26-83) | 55 | (28-85) |  | 52 | (46-62) | 54 | (49-62) | n.s |
| **Mode of detection** |  |  |  |  |  |  |  |  |  |  |  |
| Screening |  | 50 | 52.1% | 233 | 73.3% |  | 22 | (51.2) | 81 | (68.1) | n.s |
| Non- screening |  | 33 | 34.4% | 72 | 22.6% |  | 14 | (32.6) | 36 | (30.3) |  |
| Missing |  | 13 | 13.5% | 13 | 4.1% |  | 7 | (16.3) | 2 | (1.7) |  |
| **Tumor size** |  |  |  |  |  |  |  |  |  |  |  |
| < 25mm |  | 37 | (38.5) | 192 | (60.4) |  | 11 | (25.6) | 60 | (50.4) | p<0.001 |
| ≥ 25 mm |  | 46 | (47.9) | 86 | (27.0) |  | 24 | (55.8) | 41 | (34.5) |  |
| Missing |  | 13 | (13.5) | 40 | (12.6) |  | 8 | (18.6) | 18 | (15.1) |  |
| **Margin status** |  |  |  |  |  |  |  |  |  |  |  |
| Negative |  | 82 | 85.4% | 305 | 95.6% |  | 34 | (79.1) | 111 | (93.3) | p<0.001 |
| Positive/uncertain |  | 12 | 12.5% | 9 | 2.8% |  | 8 | (18.6) | 7 | (5.9) |  |
| Missing |  | 2 | 2.1% | 4 | 1.6% |  | 1 | (2.3) | 1 | (0.8) |  |
| **Microinvasion** |  |  |  |  |  |  |  |  |  |  |  |
| No |  | 89 | 92.7% | 308 | 96.9% |  | 40 | (93.0) | 110 | 92.4 | p=0.02 |
| Yes/Suspected |  | 7 | 7.3% | 10 | 3.1% |  | 3 | (7.0) | 9 | 7.6 |  |
| **Treatment** |  |  |  |  |  |  |  |  |  |  |  |
| BCS |  | 30 | 31.2% | 132 | 41.5% |  | 12 | (27.9) | 33 | (27.7) | p<0.001 |
| BCS+RT |  | 27 | 28.1% | 99 | 31.1% |  | 12 | (27.9) | 41 | (34.5) |  |
| Mastectomy |  | 39 | 40.6% | 87 | 27.4% |  | 19 | (44.2) | 45 | (37.8) |  |
