# Supplementary material for: Stromal collagen IV expression and risk of breast cancer death in ductal carcinoma in situ
Source: BJC Rep. 2025 Oct 21;3:73. doi: 10.1038/s44276-025-00191-w (PMC12540875; doi:10.1038/s44276-025-00191-w)
Supplement: Supplementary file 3 — Table S3 coll IV [file 44276_2025_191_MOESM3_ESM.docx]

Table S2: Crosstabulation of stromal and periductal collagen IV scores (maximum values) for the 17 cases assessed for expression of collagen IV in normal tissue samples within the cohort.

|  | **Stromal collagen IV** | | | |
| --- | --- | --- | --- | --- |
| **Periductal collagen IV** | 0 | 1 | 2 | 3 |
| 0 | - | - | - | - |
| 1 | 4 | 5 | - | - |
| 2 | 1 | 5 | 1 | - |
| 3 | - | - | 1 | - |
