# Supplementary material for: Stromal collagen IV expression and risk of breast cancer death in ductal carcinoma in situ
Source: BJC Rep. 2025 Oct 21;3:73. doi: 10.1038/s44276-025-00191-w (PMC12540875; doi:10.1038/s44276-025-00191-w)
Supplement: Supplementary file 4 — Table S4 coll IV [file 44276_2025_191_MOESM4_ESM.docx]

**Table S3 Stromal (a) and periductal (b) collagen IV in ductal carcinoma in situ in original four-grade scale**

**a)**

| **Stromal collagen IV** | **Cases (n=43)** | **Controls (n=119)** |
| --- | --- | --- |
| **0** | 5 (12%) | 25 (21%) |
| **1** | 18 (42%) | 62 (52%) |
| **2** | 17 (40%) | 24 (20%) |
| **3** | 2 (5%) | 7 (6%) |
| **Missing** | 1 (2%) | 1 (1%) |

**b)**

| **Periductal collagen IV** | **Cases (n=43)** | **Controls (n=119)** |
| --- | --- | --- |
| **0** | 1 (2%) | 8 (7%) |
| **1** | 16 (37%) | 56 (47%) |
| **2** | 18 (42%) | 42 (35%) |
| **3** | 8 (19%) | 13 (10%) |
| **Missing** | - | - |
