# Supplementary material for: Stromal collagen IV expression and risk of breast cancer death in ductal carcinoma in situ
Source: BJC Rep. 2025 Oct 21;3:73. doi: 10.1038/s44276-025-00191-w (PMC12540875; doi:10.1038/s44276-025-00191-w)
Supplement: Supplementary file 5 — Tables S5a_S5b [file 44276_2025_191_MOESM5_ESM.docx]

**Table S4 Collagen IV expression (four grade scale) and risk of breast cancer death.**

| **Stromal collagen IV** | OR (95% CI) |
| --- | --- |
| 0 | 1.0 (ref.) |
| 1 | 1.42 (0.46-4.39) |
| 2 | 4.00 (1.17-13.69) |
| 3 | 1.43 (0.21-9.55) |
| **Periductal collagen IV** |  |
| 0 | 1.0 (ref.) |
| 1 | 2.91 (0.29-29.37) |
| 2 | 4.22 (0.44-40.42) |
| 3 | 8.71 (0.78- 97.62) |

*OR: odds ratio; CI- confidence interval. All analyses adjusted for time at risk*
