# Supplementary material for: Stromal collagen IV expression and risk of breast cancer death in ductal carcinoma in situ
Source: BJC Rep. 2025 Oct 21;3:73. doi: 10.1038/s44276-025-00191-w (PMC12540875; doi:10.1038/s44276-025-00191-w)
Supplement: Supplementary file 6 — Table S2 [file 44276_2025_191_MOESM6_ESM.docx]

**Supplementary table 5a**

**Immunohistochemical subtype of recurrent tumour in patients with a primary DCIS analysed for collagen IV (cases versus controls)**

|  | **Cases (n=43)**  (50 events in 43 patients) | **Controls (n=119)**  (18 events in 17 patients) |
| --- | --- | --- |
| Ipsilateral   - DCIS only - ER+/HER2- - ER+/HER2+ - ER+ /HER2 missing - ER- / HER2- - ER -/ HER2+ - ER- / HER2 missing - ER missing/HER2+ - Both missing - **Total ipsilateral ER+ *** - **Total ipsilateral HER2+*** | 2  4  0  9  1  1  3  1  8  **13/18 (72%)**  **2/7 (29%)** | 7  4  0  1  1  0  0  0  1  **5/6 (83%)**  **0 (0%)** |
| Contralateral   - DCIS only - ER+/HER2- - ER+/HER2+ - ER+ / HER2 missing - ER- / HER2- - ER -/ HER2+ - ER- / HER2 missing - Both missing - **Total contralateral ER+*** - **Total contralateral HER2+*** | 4  1  1  0  3  1  0  5  **2/6 (33%)**  **2/6 (33%)** | 1  0  0  0  0  0  1  2  **0/1 (0%)**  **0/0 (0%)** |
| Distant metastasis only   - ER+/HER2- - ER+/HER2+ - ER+ / HER2 missing - ER- / HER2- - ER -/ HER2+ - ER- / HER2 missing - Both missing - **Total ER+ *** - **Total HER2+ *** | 1  0  2  0  0  0  3  **3/3 (100%)**  **0 (0%)** | -  -  -  -  -  -  -  -  - |

** Percent of all invasive tumours with available information on ER / HER2 respectively.*

*ER – oestrogenreceptor; HER2 – human epidermal growth factor receptor 2; DCIS – ductal carcinoma in situ*

**Supplementary table 5b**

**Immunohistochemical subtype of recurrent tumour in patients with a primary DCIS analysed for stromal collagen IV (low versus high collagen group)**

|  | **Low stromal**  **collagen IV (n=110)**  (37 events in 35 patients) | **High stromal**  **collagen IV (n=50)**  (30 events in 24 patients) |
| --- | --- | --- |
| **Ipsilateral**   - DCIS only - ER+/HER2- - ER+/HER2+ - ER+ /HER2 missing - ER- / HER2- - ER -/ HER2+ - ER- / HER2 missing - ER missing/HER2+ - Both missing - **Total ipsilateral ER+*** - **Total ipsilateral HER2+*** | 5  3  0  6  1  1  1  1  5  **9/12 (75%)**  **2/6 (33%)** | 4  4  0  4  1  0  1  0  5  **8/10 (80%)**  **0 (0%)** |
| **Contralateral**   - DCIS only - ER+/HER2- - ER+/HER2+ - ER+ / HER2 missing - ER- / HER2- - ER -/ HER2+ - ER- / HER2 missing - Both missing - **Total contralateral ER+*** - **Total contralateral HER2+*** | 2  0  1  0  1  1  1  3  **1/4 (25%)**  **2/3 (66%)** | 3  2  0  0  3  0  0  2  **2/5 (40%)**  **0/5 (0%)** |
| **Distant metastasis only**   - ER+/HER2- - ER+/HER2+ - ER+ / HER2 missing - ER- / HER2- - ER -/ HER2+ - ER- / HER2 missing - Both missing | 1  0  2  0  0  0  2 | 0  0  0  0  0  0  1 |

** Percent of all invasive tumours with available information on ER / HER2 respectively.*

*ER – oestrogenreceptor; HER2 – human epidermal growth factor receptor 2; DCIS – ductal carcinoma in situ*
